# Clinical Features and Surgical Results in Harada-Ito Surgery Patients

**DOI:** 10.4274/tjo.31643

**Published:** 2018-10-31

**Authors:** Önder Ayyıldız, Fatih Mehmet Mutlu, Murat Küçükevcilioğlu, Gökçen Gökçe, Halil İbrahim Altınsoy

**Affiliations:** 1 Gülhane Training and Research Hospital, Ophthalmology Clinic, Ankara, Turkey; 2 Batıgöz Hospital, Ophthalmology Clinic, İstanbul, Turkey

**Keywords:** Superior oblique muscle palsy, trochlear nerve, excyclotorsion, Harada-Ito

## Abstract

Symptomatic excyclotorsion is an important clinical problem, especially in acquired superior oblique muscle palsy. Excyclotorsion can disrupt the fusion and cause torsional diplopia. Harada-Ito surgery (HI) is a widely used method for treating excyclotorsions. This method relieves the torsional diplopia by increasing the effect of the incyclotorsion. In this study, we aimed to report the clinical features of patients with torsional diplopia due to acquired trochlear nerve palsy and the results of HI surgery in these patients.

## Introduction

The Harada-Ito (HI) procedure is a strabismus surgical technique developed to treat torsional diplopia caused by excyclotorsion resulting from superior oblique (SO) muscle palsy. The main indication for the procedure is acquired trochlear nerve palsy following closed head injury, particularly due to traffic accidents.^1^ In acquired SO palsy, torsional diplopia occurs as a result of weakened intorsional effect and the greater extorsional effect of the inferior oblique (IO) muscle. The HI procedure is an effective surgical method, especially in cases of bilateral SO palsy with a large amount of torsion.^2^ In the original technique described by Harada and Ito^4^ in 1964, the anterior fibers are advanced anteriorly without disinsertion.^3^ In 1974, Fells modified the technique and described the form that is commonly used today.^3,5^ In this modified technique, the SO muscle tendon is bisected and the anterior fibers are disinserted and transposed anterolaterally to increase the intorsional effect.^5^ The procedure can be applied unilaterally or bilaterally, depending on amount of torsion and laterality. In 1981, Metz and Lerner described the use of an adjustable suture technique with this procedure.^6^

Although there are internationally published studies concerning HI surgery, to the best of our knowledge there is no nationally published study on this subject. The existing publications are mostly from retrospective studies and the surgical techniques are usually described in these reports in writing or illustrations.^1,2,3,4,5,6^ In this study, we aimed to report the clinical features and outcomes of HI surgery in three patients who developed torsional diplopia due to acquired trochlear nerve palsy. In addition, one of our patients was evaluated with pre- and postoperative tests together with images showing the technique in order to make this rarely practiced surgical procedure more comprehensible.

The medical records of three patients who underwent the HI procedure due to torsional diplopia were retrospectively evaluated. Written consent forms were obtained from all patients prior to surgery. Medical procedures, data collection, and all stages of the study were carried out according to the Declaration of Helsinki and ethics committee approval was obtained.

For all patients, detailed medical history, examination findings, and the etiology, clinical presentation, and duration of disease were recorded. Visual acuity, anterior and posterior segment findings, eye movements, angle of deviation, abnormal head positions, oblique muscle functions, and amount of torsion were assessed pre- and postoperatively. Deviation was measured with a prism cover test, torsion was measured by double Maddox rod test, and extraocular muscle function was assessed using a Hess screen. Diagnosis of SO palsy was made based on limited depression on adduction, IO hyperfunction, V-pattern, hypertropia, abnormal head position, excyclotorsion findings, and medical history. Patients who had symptoms for at least six months underwent surgical treatment.

All patients underwent Fells’ modified HI procedure. A conjunctival incision was made in the superotemporal quadrant 8 mm from the limbus, the superior rectus (SR) muscle was isolated and the SO muscle was exposed from the lateral side. The SO tendon was split longitudinally 10 mm posterior from the insertion. The anterior fibers were separated from the insertion by suspending with 6.0 vicryl suture. The lateral rectus (LR) muscle was then isolated using a muscle hook and the anterior fibers of the SO tendon were sutured to the sclera adjacent to the superior margin 8 mm posterior to the LR insertion.

## Case Reports

The records of three patients were retrospectively reviewed in this study (Table 1). All of the patients had torsional and vertical diplopia. The etiology in all cases was closed head trauma due to vehicular accident. Based on examination findings, two patients were diagnosed with SO palsy due to bilateral trochlear nerve injury and the other patient due to unilateral trochlear nerve injury. HI surgery was performed on the affected eyes to treat diplopia.

### Case 1

**Figure 1:**
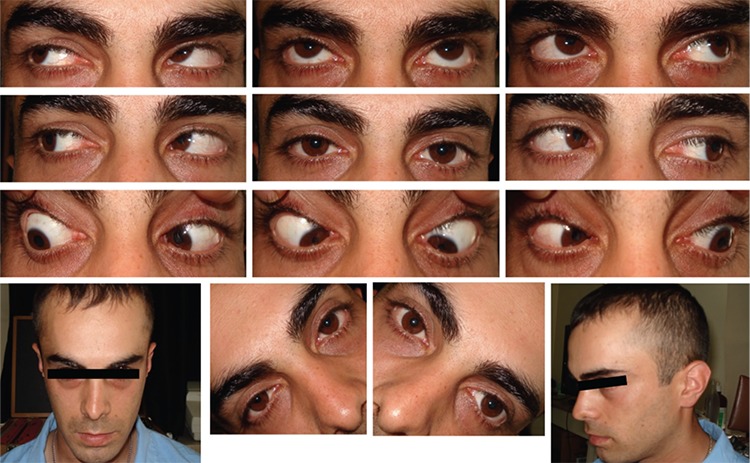
Preoperative images of patient 1. The patient had chin down head position and minimal V-pattern esotropia. Depression in adduction was -1 limited in the right eye and -2 limited in the left eye. There was +1 inferior oblique hyperfunction in both eyes

**Figure 2:**
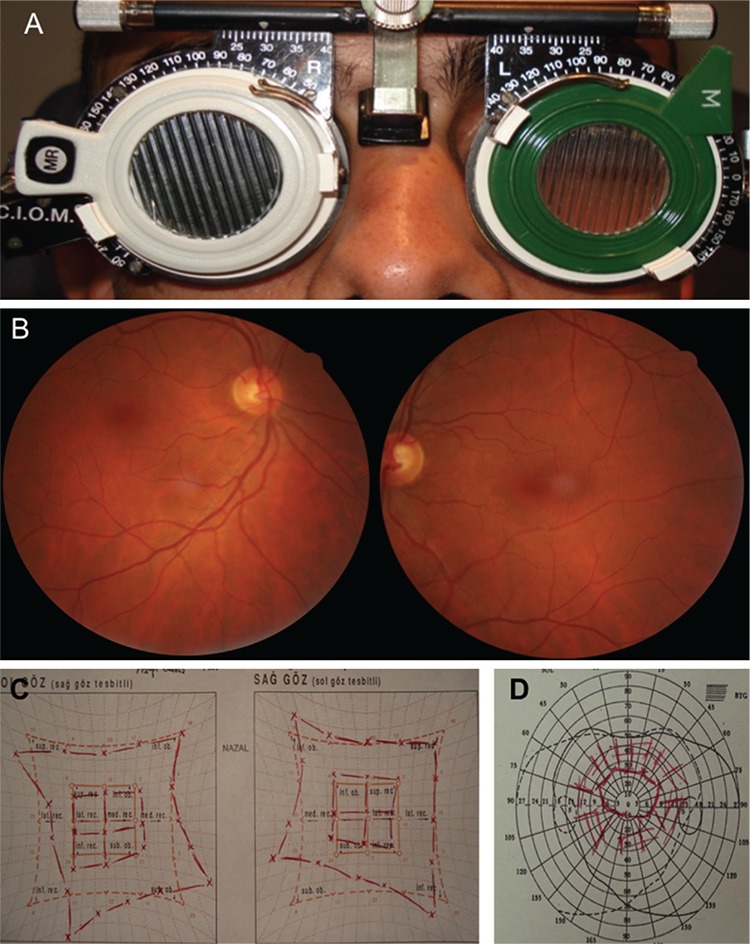
Preoperative test results in patient 1. The patient exhibited 20 degrees of extorsion in the double Maddox rod test (A) and +3 extorsion was observed in fundus images (B). Bilateral superior oblique muscle hypofunction was observed in Hess screen testing (C) and binocular visual field test revealed single vision in the superior visual field (D)

**Figure 3:**
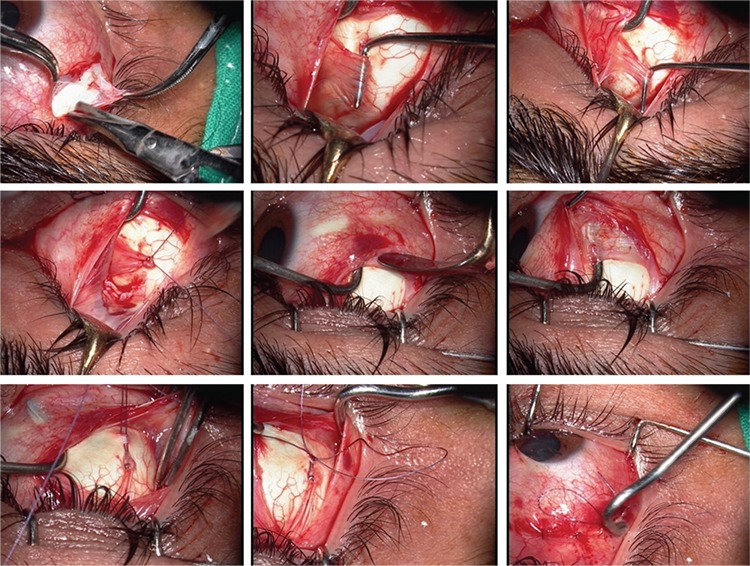
Modified Harada-Ito procedure with adjustable suture technique

**Figure 4:**
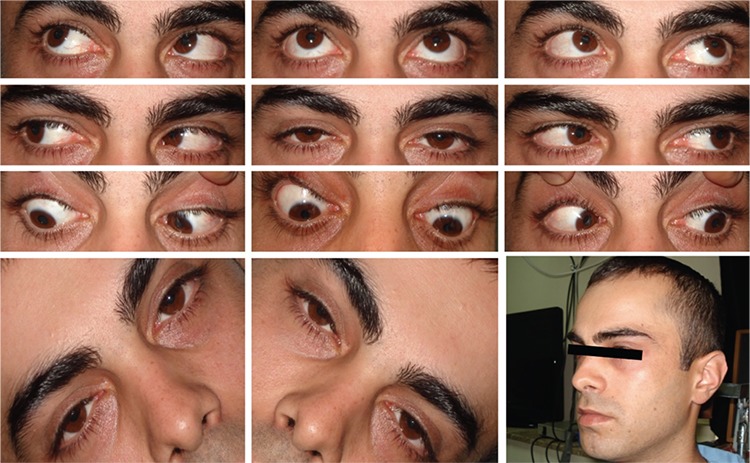
Images of patient 1 at postoperative month 4. The patient is orthotropic in primary gaze with normal head position and no torsion or limitation in the gaze positions

**Figure 5:**
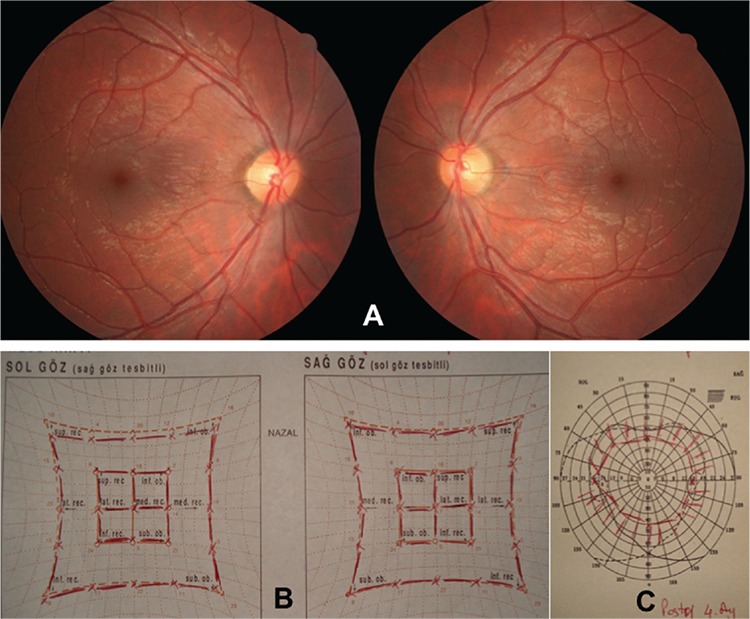
Test results of patient 1 at postoperative month 4. No torsion was observed in fundus images (A). Extraocular muscle functions were normal in Hess screen testing (B) and the area of single vision had expanded in binocular visual field testing (C)

A 28-year-old male patient presented with diplopia that was more pronounced in downgaze and had developed after a motorcycle accident a year earlier. The patient exhibited a chin-down head position and had 20/20 visual acuity and normal anterior and posterior segment examination findings in both eyes. He had minimal V pattern esotropia with -1 limited depression in adduction on the right and -2 limited depression in adduction on the left, and +1 IO hyperfunction bilaterally (Figure 1). Double Maddox rod test revealed 20 degrees of extorsion (Figure 2A) and fundus photograph revealed +3 extorsion (Figure 2B). Bilaterally reduced SO muscle function was observed on Hess screen test (Figure 2C), while binocular visual field test revealed single vision in the superior visual field (Figure 2D). Based on ophthalmic examination findings, the patient was diagnosed with bilateral SO palsy and underwent modified HI with adjustable suture technique in the right eye and modified HI procedure in the left eye (Figure 3). On postoperative day 1, double Maddox rod test revealed 5 degrees of extorsion and the suture was adjusted to eliminate this remaining torsion. On postoperative day 3, the patient’s head position was improved, he was orthotropic in primary gaze, and fundus photography showed +1 intorsion. At postoperative 4 months, the patient was orthotropic with no limitation or torsion in any gaze position, and maintained straight gaze (Figure 4). There was no torsion in fundus images. Extraocular muscle functions were normal in the Hess screen test and his field of single vision in binocular visual field testing had expanded (Figure 5).

### Case 2

A 53-year-old male patient presenting with diplopia stated that his complaint had started after a traffic accident 6 months earlier. He had 20/20 vision in both eyes and normal biomicroscopic and fundoscopic examination findings. The patient exhibited a chin down head position and had torsional diplopia as well as V-pattern esotropia of 12 prism diopters on downgaze. He had limited depression in adduction (-2) in both eyes, but no IO hyperfunction. Double Maddox rod test revealed 20 degrees of extorsion and fundus photography revealed +2 extorsion. Bilateral SO muscle hypofunction was observed in Hess screen test and binocular visual field testing revealed diplopia on downgaze. The patient was diagnosed with bilateral SO palsy based on examination findings, and the modified HI surgery was performed in both eyes. At postoperative week 1, the patient showed improved head position, fundus images showed no extorsion, and 2 degrees of extorsion were observed in the double Maddox rod test. Hess screen test showed normal SO muscle function bilaterally and slight IO hypofunction. Diplopia on downgaze was not detected in binocular visual field testing. At postoperative 4 months, the patient exhibited normal head position and was orthotropic in primary gaze. No torsion was observed in double Maddox rod test and fundus images. The patient described slight diplopia on upgaze. Hess screen test showed normal SO function with -1 hypofunction in the IO muscles. No additional intervention was done.

### Case 3

A 58-year-old female patient reported developing double vision following a traffic accident 1 year earlier, and that later her right eye gradually developed an upward deviation. Her vision was 20/20 in both eyes and her anterior segment examination and fundoscopy findings were normal. She exhibited a left head tilt. In primary gaze position, hypertropia of 14 prism diopters at distance and 12 prism diopters at near was measured in the right eye. Depression in adduction was -2 limited and IO hyperfunction was not observed in the right eye. Double Maddox rod test revealed 10 degrees of extorsion and +2 extorsion was measured on fundus photography of the right eye. Hess screen test revealed reduced SO muscle function in the right eye and binocular visual field testing revealed diplopia on downgaze. She was diagnosed with right SO palsy and modified HI surgery with 5.5-mm SR recession was performed. At postoperative 1 week, the patient showed improved head position and extorsion. Minimal hypertropia was observed on the right eye in primary gaze position, while Hess screen test revealed improved SO muscle function in the right eye and binocular visual field testing demonstrated reduction in the area of diplopia on downgaze. Examination findings at the first postoperative month showed no further changes, and the patient continued follow-up in a different city.

## Discussion

The long intracranial course of the trochlear nerve makes it especially prone to injury in closed head traumas. Acquired trochlear nerve palsy may cause symptomatic excyclotorsion, also referred to as torsional diplopia, which is rarely seen in congenital cases.^7^ Managing patients with this complaint is difficult. The main goal of surgical treatment in these patients is to provide single vision in primary and downgaze and to correct abnormal head position.^7^ While various surgical techniques have been described for the treatment of SO palsy due to acquired trochlear nerve injury, the modified HI procedure is very effective in reducing excyclotorsion and treating torsional diplopia.^7^In the modified HI technique, the anterior fibers of the SO muscle are transposed anterotemporally, strengthening the intorsion effect of the muscle.^2^

In our study, HI surgery was successfully performed on five eyes of three patients with torsional diplopia. Postoperative diplopia was not observed with distant or near fixation in the primary gaze position. However, surgical success rates of 43-68% have been reported in previous studies.^2,3^ Preoperatively, our patients exhibited abnormal head positions they had developed to prevent diplopia and achieve fusion. We found that in all cases, head position was improved and fusion achieved postoperatively. Bradfield et al.^8^ demonstrated that the presence of fusion prior to surgery was associated with surgical success.

Another factor determining surgical success is preoperative amount of torsion. Bradfield et al.^8^ reported that surgical success increased as the amount of torsion decreased. Torsion usually does not exceed 10 degrees in unilateral SO palsy; as in our third case, patients with complaints of diplopia occurring immediately after head trauma later report unilateral hypertropia.^1^ In these patients, SR recession or IO muscle weakening to correct hypertropia can be performed concurrently with the HI procedure to correct torsion. Our third patient, whose right eye was hypertropic in addition to having 10 degrees of torsion, underwent HI surgery and concurrent SR recession due to the absence of IO hyperfunction. Patients with more than 10 degrees of excyclotorsion are usually symptomatic, as in our other 2 cases. Such patients have a chin down head position, and bilateral SO muscle palsy should be suspected.^2^ Together with a history of head trauma, examination findings of V-pattern, more than 10 degrees of excyclotorsion, and left hypertropia in right gaze and right hypertropia in left gaze are suggestive of bilateral SO palsy.^1^ Managing bilateral acquired SO palsy may be difficult because of the considerable amount of torsion, but bilateral HI surgery can successfully reduce extorsion and alleviate symptoms in these patients.^2^ To improve the success of surgical treatment, the HI procedure should be used in patients with primary complaints of torsion in particular.

Taking into account coexisting vertical and horizontal deviations, V-pattern, and IO hyperfunction during surgical planning influences surgical success. Tendon transposition of the rectus muscles, inferior or superior rectus recession, and IO muscle weakening surgery to correct V-pattern can also be performed together with the HI procedure.

As with our second patient, there are patients who postoperatively develop limitation of movement in the IO muscle field and diplopia on upgaze, similar to Brown’s syndrome.^8^ Patients undergoing surgery should be informed about complications such as iatrogenic restriction and under- or overcorrection. As in our first case, intorsion may develop in the early postoperative period following HI surgery for symptomatic extorsion. Intorsion usually regresses during follow-up. Postoperative recurrence of symptomatic extorsion has been reported in several studies; therefore, an overcorrection of up to 10 degrees of intorsion is recommended.^2,3,9^ Residual excyclotropia occurring later in the postoperative period, as in our second case, is common.^1,3,9^ It has been reported that patients with acquired cyclotropia exhibit retinal sensory reorientation to overcome torsion and are only symptomatic in dissociated environments.^10^

Limitations of our study include the fact that it was a retrospective chart review. In addition, statistical analyses could not be done due to the small number of patients and paucity of data, and follow-up was short.

In conclusion, HI surgery successfully treated torsional diplopia, especially in primary gaze position, in all three of our patients. Preoperative amount of torsion and the presence of fusion can affect surgical success. Prior to surgery, patients should be informed that diplopia may persist postoperatively, especially in downgaze, that this may necessitate an additional intervention or the use of prisms, and that iatrogenic Brown’s syndrome may develop and cause diplopia in upward gaze.

## Figures and Tables

**Table 1:**
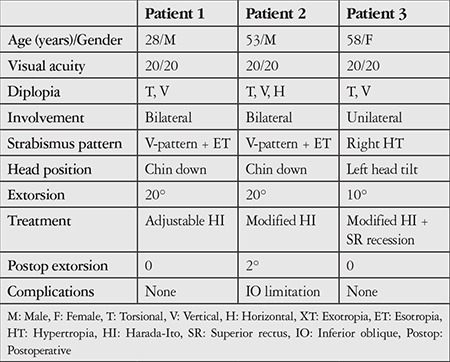
Pre- and postoperative characteristics of the patients
